# Classification system for nanotechnology-enabled health products with both scientific and regulatory application

**DOI:** 10.3389/fmed.2023.1212949

**Published:** 2023-07-14

**Authors:** Francisco D. Rodríguez-Gómez, Oriol Penon, Dominique Monferrer, Pilar Rivera-Gil

**Affiliations:** ^1^Asphalion SL, Barcelona, Spain; ^2^Integrative Biomedical Materials and Nanomedicine Lab, Department of Medicine and Life Sciences, Universitat Pompeu Fabra Barcelona Biomedicine Research Park (PRBB) Doctor Aiguader, Barcelona, Spain

**Keywords:** regulatory science, regulatory uncertainties, nanomedicines, nanotechnology enabled health products, classification

## Abstract

The lack of specific regulatory guidelines for nanotechnology-enabled health products (NHPs) is hampering development and patient access to these innovative technologies. Namely, there is an urgent need for harmonized regulatory definitions and classification systems that allow establishing a standardized framework for NHPs regulatory assessment. In this work, a novel classification system for NHPs is proposed. This classification can be applied for sorting nano-based innovations and regulatory guidelines according to the type of NHPs they address. Said methodology combines scientific and regulatory principles and it is based on the following criteria: principal mode of action, chemical composition, medical purpose and nanomanufacturing approach. This classification system could serve as a useful tool to sensor the state of the art of NHPs which is particularly useful for regulators to support strategy development of regulatory guidelines. Additionally, this tool would also allow manufacturers of NHPs to align their development plans with their applicable guidelines and standards and thus fulfill regulators expectations.

## 1. Introduction

Nanomedicine can be considered as the field where nanoscience interacts with life for the development of nanotechnology-enabled health products (NHPs) (1)^1^. This area has become one of the most promising disciplines of the 21st century. In fact, the market size of NHPs had an estimated value of 53 billion United States Dollars (USD) in 2009 and a predicted projection of approximately 334 billion USD by 2025 (around a 630% increase) (2).

Despite scientific and economic interests, there is still a considerable gap between the number of NHPs in research and development and those eventually reaching society. This becomes more evident when comparing the number of scientific publications or patents (NHPs at an early-medium stage of research and development) and the number of clinical studies with NHPs (NHPs reaching the latest stages of clinical development which may be closed to regulatory approval) with the number of approved NHPs.

**Figure 1:**
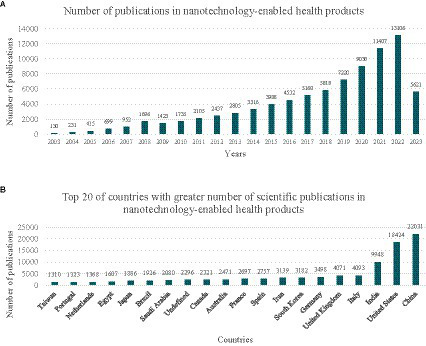
Analysis of literature search for nanonotechnology-enabled-healthcare-product-related manuscripts. **(A)** It shows the number of publications referring to NHPs has always followed an increasing trend over the last 20 years. **(B)** Regarding the top 20 countries with the highest number of scientific publications, EEA, China and the USA, are the regions with the highest number of publications in ascending order. Scopus database, literature search carried out in May 2023 by using search string ‘nanomedicine OR “nanomedical device” OR (nano* AND health)’.

Over the last 20 years, the number of scientific references in NHPs under search string <<nanomedicine OR “nanomedical device” OR (nano* AND health)>> in scientific literature repository Scopus has considerably increased up to a total of 83,826 results as of May 2023 (Figure 1A). Most specifically, China, the United States of America (USA) and the European Economic Area (EEA) are positioned as the geographical regions in the *top 20* with the highest number of publications worldwide (22,031, 18,424 and 15,736 respectively) (Figure 1B). With regards to patents, 36,864 results appear in the World Intellectual Property Organization (WIPO) patent repository under keyword ‘*nano*’ by using the following search string: <<EN_TI:(nano*) AND CPC^2^*:(A61) AND DP:([01.01.2003 TO 31.05.2023])*>> (results including root ‘nano*’ in the title over the period between January 2003 and May 2023 and that are listed under CPC code A61 applicable for medical or veterinary science, hygiene).

**Figure 2:**
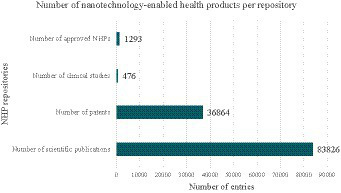
Valley of death in nanotechnology-enabled health products (NHPs) development. The number of nanotechnology-enabled health products (NHPs) at an early-medium stage of research and development is represented by the number of scientific publications and patents related to NHPs. Additionally, the number of clinical studies with NHPs is a marker of the number of applications reaching the latest stages of clinical development, close to regulatory approval. When comparing both markers with the number of NHPs currently in use, a valley of death can be noticed.

As for clinical studies, there are around 476 entries in the ClinicalTrials.gov database under the keyword ‘*nano*’ with <<*study start date*>> in the last 20 years. Additionally, NHPs currently placed on the market, meaning that have reached regulatory approval, have also been considered to have a complete picture of NHPs in different stages of health product lifecycle. This is such a complicated task since there is no official repository of all NHPs currently marketed globally. Notwithstanding, the Nanotechnology Product Database (NPD) has been taken into account. This directory is regarded as one of the most reliable tools for institutions and policymakers involved in the establishment of their country policies and national strategic plans (3). According to the NPD, there are 1,293 NHPs currently marketed with medical applications. When compared to the number of scientific publications, patents, the ratio of references versus approved NHPs is appreciably high (Figure 2). The difference between the number of NHPs early in development (scientific publications and patents) and which have reached regulatory approval (approved NHPs) is commonly referred to as the *valley of death* (4–6). According to different authors, this scenario is caused due to methodological barriers for a better characterization and understanding of NHP performance. Additionally, while regulatory agencies have setup guidelines to support and smooth NHP translation from bench to bedside, the gap is still hardly overcome by NHPs manufacturers and developers (4, 7).

To mitigate this current situation, regulations and technological progress must advance in tandem to ensure derived innovations can be safely and effectively assessed by the corresponding regulatory authorities. Indeed, highly disruptive, and complex products, such as NHPs, need a clearer regulatory pathway to reach the patients. Notwithstanding, regulatory science^3^ faces important challenges to develop standards and guidelines applicable to nanotechnological breakthroughs to facilitate their translation into society (8, 9). Proof of this is the absence of harmonized regulatory definitions of key terms such as nanomaterials, nanotechnology, nanopharmaceutical, or nanomedicine and classification systems for NHPs, which is a relevant limitation for the interoperability of the different stakeholders (regulators, academic researchers, physicians, and the industry) (10, 11).

**Figure 3:**
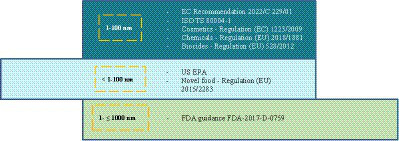
Materials classified as nanomaterials depending on the applicable legislative framework or regulatory guidance.

On the one hand, there are different definitions for the term ‘nanomaterial’ depending on the applicable legislative framework or regulatory guidance. The size range 1–100 nm is broadly included in the definition of nanomaterial in the USA and EEA, namely provided by the United States Environmental Protection Agency (US EPA), the Food and Drug Administration (FDA) guidance FDA-2017-D-0759 on products involving nanotechnology, the European Commission (EC) recommendation on nanomaterial definition (2022/C 229/01), International Organization for Standardization (ISO) standard ISO TS 80004–1 on nanotechnology definitions and regulations on novel food, cosmetics, chemical substances and on biocides. Despite this, there are different nuances regarding what is considered at the nanoscale. For example, no lower limit is specified by the US-EPA or the novel food regulation. Additionally, regarding FDA-2017-D-0759 guidance, the upper limit is flexible up to 1,000 nm. Finally, the EC recommendation 2022/C 229/01, the chemical substances regulation and the biocides regulation limit the applicability of the nanomaterial definition to materials for which at least 50% of their particles are within the size range of 1–100 nm (12–19). Consequently, a material would be nanomaterial or not depending on its regulatory qualification as well as depending on the geographical region, given that the corresponding applicable legislative framework considers the applicability of the nanomaterial concept slightly differently (Figure 3).

On the other hand, there are several classification systems for NHPs based on different criteria depending on the field of knowledge (20). Nonetheless, the regulatory field has no established classification system for NHPs (21). Consequently, it is ambiguous to NHPs developers, manufacturers and regulators deciding which regulatory guidelines are applicable to each NHPs. Thus, classifying both NHPs and regulatory guidance with a system based on criteria with a significant impact on the product regulatory development would allow facilitate product approval.

This work presents a novel classification system for NHPs with the aim of unlocking the development of NHP-specific harmonized regulatory guidelines that are useful for scientific and regulatory purposes.

## 2. Materials and methods

The following steps have been taken in the development of the proposed nanomaterials classification system: (i) defining key parameters, criteria, to consider, (ii) generating different NHP categories based on those parameters, and (iii) testing the proposed classification system against current developing and published regulatory guidelines.

### 2.1. Regulatory guideline compilation

Guidelines and other regulatory documents have been gathered in a regulatory database that includes references identified as of May 2023 from the following sources:

o Competent authorities:

▪ EMA:

•https://www.ema.europa.eu/en/human-regulatory/research-development/scientific-guidelines/multidisciplinary/multidisciplinary-nanomedicines

▪ FDA:

•https://www.fda.gov/science-research/nanotechnology-programs-fda/nanotechnology-guidance-documents

o EC:

▪ EU Science Hub: https://ec.europa.eu/jrc/en/research-topic/nanotechnology

▪ Scientific Committee on emerging and newly identified health risks (SCENHIR):

•https://ec.europa.eu/health/scientific_committees/emerging

o Organization for the Economic Co-operation and Development (OECD), Safety of manufactured nanomaterials:

•https://www.oecd.org/science/nanosafety/

o Standard emitting organizations:

▪ ISO, Technical committee ISO/TC 229 on Nanotechnologies: https://www.iso.org/committee/381983.html

▪ American Society for Testing and Materials (ASTM), Technical subcommittee E56.08 on Nano-enabled medical products:

•https://www.astm.org/COMMIT/SUBCOMMIT/E5608.htm

### 2.2. Data management

Databases have been generated with Excel macro-enabled workbook format (Microsoft Office Professional Plus 2019). Data processing and plotting was carried out by using dynamic tables.

## 3. Results and discussion

### 3.1. Classification criteria with scientific and regulatory implications

#### 3.1.1. Tentative legislative framework

Quality, safety, performance and efficacy requirements applicable to a health product depend on the regulatory qualification of the product, i.e., its regulatory category (medicinal product or medical device or combinations thereof). Additionally, these requirements are detailed in the legislative framework applicable to each regulatory category. Therefore, the first aspects to consider for the proposed classification system are characteristics determining the qualification of a given NHP under the regulatory categories for products for medical purposes, namely medicinal product, or medical device (i.e., the tentatively applicable legislative framework).

The main aspects characterizing medicinal products and medical devices regulatory categories are:

- The *purpose of the technology*, the product shall be intended to be used for one or more specific medical purposes in humans. This aspect is common for medicines and medical devices regulatory categories at the EEA and the USA.
- The *primary mode of action* by which the technology achieves its medical purpose, i.e., whether the product exerts its principal action by pharmacological, immunological or metabolic means or not (1, 22). If the product achieves the intended action by such means it is considered a medicinal product, if not, it is considered a medical device. In the latter case, medical devices achieve their intended purpose by primary mechanical or physical means (which in turn can be combined with an ancillary pharmacological action). Thus, this aspect is the differentiating characteristic between medicinal products and medical devices.

For this reason, the primary mode of action is the first criterion in the proposed classification system, which addresses the following questions: *does the product exert its primary mode of action based on pharmacological, immunological, or metabolic means*? For the purposes of this classification system, the application of this criterion does not imply that the NHP have been classified as a medicinal product or a medical device as the stage of development of the assessed technologies might be too immature to perform a regulatory qualification.

#### 3.1.2. Quality and safety of NHPs

Safety and overall benefit–risk- ratio assessment are important aspects for obtaining regulatory approval of all health product regulatory categories. In the case of NHPs, as well as for other technologies, risk assessment shall strongly rely on a thorough physicochemical characterization, which is, in turn, closely linked to the safety and performance of the product (4, 23–25). Additionally, physicochemical properties can impact the absorption, distribution, metabolism and elimination of NHPs in the human body. Therefore, it is considered essential to include physicochemical characterization as a criterion of the proposed classification system.

Due to nanomaterials’ great complexity, there is no harmonized ‘list of physicochemical parameters’ to be taken into account for defining a characterization plan of NHPs that meets regulators expectations (26–28). Currently, there are several expert groups (Table 1) working on how to better conduct nanomaterials’ physicochemical characterization. In this work, available specific regulatory guidelines addressing characterization of NHPs have been considered to define which physicochemical parameters would better suit as criterion for the proposed classification system (42).

**Table 1:** Expert working groups on manufactured nanomaterials (non-exhaustive list).

Expert working groups in NHP	Type of entity	Reference
ASTM E56: Nanotechnology	Standards issuing organization (USA)	(29)
ISO/TC 229: Nanotechnologies	Standards issuing organization (international)	(30)
EU Nanotechnology Characterization Laboratory (EU-NCL)	Testing laboratory	(31)
National Cancer Institute (NCI) Nanotechnology Characterization Laboratory (NCL)	Testing laboratory	(32)
NCI Nanotechnology Working Group (Nano WG)	Working group (USA)	(33)
Working Party on Manufactured Nanomaterials (WPMN)	Working group (international)	(34)
Safe-N-Medtech	EU funded project (European Commission)	(21)
Refine Nanomed	EU funded project (European Commission)	(22)
European Technology Platform Nanomedicine (ETPN)	Organization (Europe)	(35)
Nanomedicines Working Group (NWG) of the International Pharmaceutical Regulators Programme (IPRP)	Working group (international)	(36)
European Federation of Pharmaceutical Sciences (EUFEPS)	Organization (Europe)	(37)
Lygature	Working group (international)	(38)
European Foundation for Clinical Nanomedicine (CLINAM)	Foundation (Europe)	(39)
Global Coalition for Regulatory Science Research (GCRSR)	Conference for discussion (international)	(40)
EDQM’s Working Party on Non-Biological Complexes (NBC)	Working group (international)	(41)

Regarding medicinal products, numerous general and specific guidelines have been published by the EMA and the FDA. Furthermore, the European and American Pharmacopeias (Ph. Eur. and USP) include chapters and monographs with standardized methods for the assessment of quality parameters of medicinal products, which in most of the cases do not address specifically NHPs (7, 43). In the EEA, the EMA issued a series of four reflection papers on nanomedicines and nanosimilars to provide guidance to manufacturers during the development of specific products, namely liposomal formulations (44), block-copolymer-micelles (45), iron-based nanocolloids (46) and coated nanomedicine products (47). Additionally, in the USA, the FDA published six guidelines related to the application of nanotechnology in different regulatory categories (e.g., novel food, drugs, or cosmetics). Most specifically, regarding medical product regulatory categories, a guidance on liposome drug products (48) and a general guidance on drug products containing nanomaterials (49) are available.

As to medical devices, developers and manufacturers shall demonstrate compliance with applicable quality requirements based on any of the following ways: following applicable technical standards or common specifications (where available), setting up own methods or referring to relevant published literature. In order to bring some light to this issue, EC published a guidance addressing specific parameters for the safety evaluation of nanomaterials used in medical devices (50). Furthermore, guidance on the physicochemical characterization of manufactured nano-objects is provided in the standard ISO/TR 13014:2012(E): *Guidance on physico-chemical characterization of engineered nanoscale materials for toxicological assessment* (51). This international technical report, although it is not specific to the medical device regulatory category, is considered not applicable for medicinal products.

Physicochemical characterization parameters addressed in the above-mentioned guidance documents have been listed in Table 2. Based on this table, it can be concluded that general physicochemical parameters defined in guidelines issued by EMA, EC and the FDA can be broadly mapped within the ISO/TR 13014:2012(E) In other words, ISO/TR 13014:2012(E) despite not being applicable for medicinal products, can be considered as a reference standard on physicochemical parameters for NHP characterization.

**Table 2:** Mapping of physicochemical characterization parameters established by EMA nanomedicines guidelines in ISO/TR 13014:2012(E).

		EMA/CHMP/SWP/100094/2011 (46)	EMA/CHMP/806058/2009/Rev. 02 (44)	EMA/CHMP/13099/2013 (45)	EMA/325027/2013 (47)	FDA guidance – Liposome Drug Products (52)	FDA guidance – Drug products containing nanomaterials (49)	FDA publication (Zheng N., 2017) (53)
ISO/TR 13014:2012 (E): Guidance on physicochemical characterization of engineered nanoscale materials for toxicological assessment (32)	Particle size/distribution	Particle size, size distribution.	Mean size and size distribution.	Micelle size and distribution profile.	Complete characterization, including composition and control.	Particle size	Average particle size, size distribution.	Mean size and size distribution.
	Aggregation/agglomeration state in relevant media	Not described.	Aggregation.	Not described.		Not described.	Aggregation and agglomeration or separation.	Oligomeric status.
	Shape	Morphology.	Liposome morphology.	Morphology.		- Liposome morphology. - Liposome structure	General shape and morphology.	Morphology and structure.
	Surface/specific surface area	Surface properties.	Not described.	Not described.		Surface characteristic as applicable.	Surface area, ligands, hydrophobicity and roughness.	Surface properties.
	Composition	Structure and composition of carbohydrate matrix.	Not described.	Chemical structure.		Not described.	- Chemical composition. - Chemical stability	Not described.
	Purity (including levels of impurities)	Impurities.	Purity.	Not described.		Not described.	Impurities.	Impurities.
	Surface chemistry	Surface properties.	Not described.	Not described.		Not described.	Surface chemical reactivity.	Surface properties.
	Surface charge	Charge.	Not described.	Zeta potential.		Net charge.	Surface charge.	Surface charge or Zeta potential.
	Solubility	Not described.	Not described.	Not described.		Not described.	Not described.	Not described.
	Dispersibility	Not described.	Not described.	Not described.		Not described.	Not described.	Not described.
Additional specific parameters*		- Spectroscopic properties. - Polymorphic form of the iron comprising the core. - Ratio of bound carbohydrate to iron.	Fraction of encapsulated active substance.	- Other surface properties (e.g., targeting ligand). - Drug loading. - Associated number. - Viscosity. - Physical state of the active substance.	Not described.	- Drug product viscosity and parameters of the contained drug. - *In vitro* release of the drug substance. - Liposome phase transition temperature. - Leakage rate of drug from the liposomes.	- Structural attributes. - Coating properties. - Porosity. - *In vitro* release. - Coating properties. - Biodegradability - Sterility, endotoxin levels and pyrogenicity.	- *In vitro* release. - Osmolarity. - Optical structure. - Molecular. weight distribution.

^*^Additional parameters laid down in EMA guidelines are specific for the NHP they are addressing, in these cases, either with coating or carrier actions. Parameters highlighted in green are deemed as already covered in ISO/TR 13014:2012(E).

Next, the suitability as classification criterion of each of the ISO/TR 13014:2012(E) parameters is discussed (Table 3). The first fundamental aspect that has been considered is whether the parameters are qualitative (such as the composition, or the shape) or quantitative (such as the particle size, or solubility). As a rule of thumb, nanomaterials, as dynamic systems, are mostly defined by continuous quantitative variables which reflect their erratic behavior depending on the media. Nonetheless, for the purposes of developing a classification system, it could be challenging to differentiate between different categories based on quantitative variables. For instance, there is much controversy regarding the various definitions of nanomaterial that depend on a threshold of size (quantitative variable). As recognized by the EC, there is no clear scientific justification for setting the size thresholds (14, 60). Moreover, it is commonly acknowledged that many properties of materials are size-dependent and that the fixed threshold between 1–100 nm for nanomaterials was introduced for regulatory purposes, rather than for scientific reasons (61, 62). There is no well-defined transition point at which a property or its value becomes characteristic for the nanoscale, and not all materials exhibit the same phenomena (63). Consequently, quantitative variables have been discarded as criteria of the proposed classification system.

**Table 3:** Tentative physicochemical parameters considered as criterion for the proposed classification system.

Physicochemical parameter		Definition	Reason for being considered or excluded as classification criterion
Physical description	Particle size/distribution	Length of one or several specific aspects of the particle geometry (54). Particle size distribution refers to the cumulative distribution of particle concentration as a function of particle size (51).	This parameter is considered a quantitative continuous variable and it has therefore been ruled out as a possible criterion for the proposed classification system.
	Aggregation/agglomeration state in relevant media	Number and distribution of aggregate/agglomerate particles in comparison to the total number of primary particles (54).	This parameter is considered a quantitative continuous variable and it has therefore been ruled out as a possible criterion for the proposed classification system.
	Shape	Description of the contour or outline surface of a nanomaterial, collection of nanomaterials, aggregates or agglomerates (51).	Different shapes can lead to different nanomaterials effects on cellular internationalization, differentiation, etc. However, the number of possible architectures is very large and new possibilities are being explored all the time (55, 56). Moreover, this aspect is also affected by the number of nanodimensions that a nanomaterial presents, and not all architectures are available for all types of dimensions. Thus, in order to create a classification system with ‘populable’ categories, it is interesting not to generate subdivisions that although highly specific would scarcely be regulatorily significant. For this reason, this criterion has been ruled as applicable for the proposed classification system.
	Surface area/specific surface area	Surface area is the quantity of accessible surface of a nanomaterial when exposed to either a gaseous or liquid adsorbate phase. It is conventionally expressed as a mass specific surface area or as volume specific surface area where the total quantity of the area has been normalized either to the nanomaterial’s mass or volume, respectively (51).	This parameter is considered a quantitative continuous variable and it has therefore been ruled out as a possible criterion for the proposed classification system.
Chemical composition	Composition	Property given by the identity and content of each specific chemical component of a nanomaterial (can be expressed as a chemical formula) (51).	Chemical composition governs nanomaterial’s optical, magnetic, catalytic and toxicological characteristics (57, 58). Nanomaterials dispersed in a solvent, such as biological media, form colloidal systems. Depending on the chemical nature of the discontinuous phase, i.e., the nanomaterial, the colloidal system will exhibit a specific biological behavior (59). Thus, this physicochemical characterization parameter is selected as a parameter of interest to be applied as a criterion in the proposed classification system.
	Purity	Level or concentration of unintended constituents (impurities) (54).	This parameter is considered a continuous variable and it has therefore been ruled out as a possible criterion for the proposed classification system.
	Surface chemistry	Chemical nature, including composition, of the outermost layers of the nanomaterial (51).	It is possible in some cases that the molecules attached to the surface could be considered under ‘composition’ `parameter; however, the preference is to characterize surface chemistry separately (51). In turn, nanomaterials, as dynamic, erratic systems, may differ depending on the surrounding media. Consequently, surface chemistry not only depends on the characteristics of the NHP but also of the characteristics of the constituents present in the media where the NHP is dispersed (26). Consequently, this physicochemical characterization parameter has been ruled out as a possible criterion for the proposed classification system.
Extrinsic properties	Surface charge	Electrical charge on a surface (51).	This parameter is considered a continuous variable and it has therefore been ruled out as a possible criterion for the proposed classification system.
	Solubility	Maximum mass of a nanomaterial that is soluble in a given volume of a particular solvent under specified conditions (51).	This parameter is considered a continuous variable and it has therefore been ruled out as a possible criterion for the proposed classification system.
	Dispersibility	Level of dispersion when it has become constant under the defined conditions (45).	This parameter is considered a continuous variable and it has therefore been ruled out as a possible criterion for the proposed classification system.

As concluded from Table 3, the chemical composition of NHPs is selected as applicable criterion of the proposed classification system. This parameter is understood as the predominant chemical identity of the core of a nanoparticle, and it is therefore considered a qualitative variable. Furthermore, composition is described based on descriptive categories (i.e., chemical identities) expressed as a chemical formula, crystalline state, molecule structure-conformation (51). Consequently, this criterion will be addressing the following question: *what is the chemical composition of the NHP?* This question shall have a limited number of answers (previously referred to as categories). These accepted answers have been defined based on ISO/TR 11360:2010: *Nanotechnologies — Methodology for the classification and categorization of nanomaterials* (64). This technical report describes five different categories for classifying nanomaterials by chemical identity (ceramic, metallic, semi-metallic, polymer [natural/synthetic] and carbon based). Additionally, these categories can be further clustered into three groups with similar biological behavior. This approximation is based on the Staudinger classification according to atomic binding of colloidal systems (wherein the dispersing phase is the nanomaterial [particles of 10^3^ to 10^9^ atoms]) (59):

- *Dispersoid colloids*: nanomaterials made up of inorganic elements (such as ceramic, metallic, or semi-metallic) which are dispersed in a solvent. They are thermodynamically unstable and lyophobic (hydrophobic colloids).
- *Molecular colloids*: nanomaterials made up of all types of polymers (both natural and artificial) and that are dispersed in a solvent. They are thermodynamically stable and lyophilic.
- *Associated colloids*: nanomaterials made up of amphipathic molecules (mainly carbon-based molecules) that self-assemble in the presence of a solvent and a tension-active surfactant. They are thermodynamically unstable and lyophilic.

#### 3.1.3. Pre-clinical and clinical assessment

Another general requirement for the regulatory assessment of health products is the demonstration of the mode of action of the NHP and the evaluation of its efficacy based on *in vitro*, animal and/or human studies.

The regulatory strategy to address performance and efficacy of NHPs and the parameters to be assessed will depend on the medical use, including clinical indication and attributed benefits or claims. Therefore the next criterion considered for the proposed classification system is the broad medical use of the NHP. The question that this criterion would address is: *what is the medical use of the NHP*?

On the one hand, for medicinal products, the definitions given by the EMA and the FDA include products with diagnostic, preventive or treating actions. In addition, apart from the active pharmaceutical ingredients (APIs), medicinal products also include excipients which, by definition, are the constituents of the pharmaceutical form apart from the API that can modify the pharmacokinetic profile of the formulated active substance (65). On the other hand, the definition of medical device includes products that have diagnostic or therapeutic (including treatment, compensation, alleviation, prevention, and monitoring) actions.

Based on the different possible general uses (diagnosis, preventive, treatment, or galenic purposes), the following categories are proposed for this criterion: (i) pharmacological therapeutic action, (ii) pharmacological prophylactic action, (iii) non-pharmacological therapeutic (treating/prophylactic) action, (iv) diagnostic action (either *in vitro* or *in vivo*) and (v) technological or galenic action.

#### 3.1.4. Sustainability of NHPs

Environmental pollution caused by pharmaceutical substances is an emerging environmental problem. In 2013, the BIO Intelligence Service (the European Environmental Agency) published a study on the risks of environmental effects of medicinal products. According to this study, residues of various types of health products have been detected in various environmental compartments, such as surface water, groundwater, soil, air and biota (66, 67). This environmental accumulation of pharmaceutical substances poses an environmental hazard that has been broadly recognized. Consequently, the hazards and risks posed by residues from NHP manufacturing (nanomanufacturing) cannot be underestimated (68). In this sense, green nanotechnology is an emerging field for the implementation of sustainable principles for the manufacture of NHPs (69, 70).

Bearing this in mind, regulatory measures have been put in place to control environmental risk during health products development and manufacturing. At EEA level, for medicinal products all new marketing authorization applications are required to undergo an environmental risk assessment following a tiered assessment procedure (71). In the case of medical devices, Regulation 2017/745 and Regulation 2017/746 include a general requirement for safe disposal of the product and safe disposal of related waste substances by the users (72, 73).

Focusing on NHPs, there are mainly two approaches for the nanomanufacturing: top-down (nanomaterials are synthesized by breaking bulk materials into smaller pieces) and bottom-up (nanomaterials are synthesized from atomic or molecular species) (74). As a general rule, the top-down approach results in the production of more waste than the bottom-up (75). In fact, the bottom-up approach allows customization of the design of reactions and can be based on biological reactions for nanomanufacturing (like microorganisms, enzymes, molecules), therefore following a green nanomanufacturing approximation (76, 77). Additionally, broadly speaking, green nanomanufacturing exhibits interesting properties such as nontoxic, cost-effective, biodegradable, energy-efficient, and enhanced-biocompatibility. For all these reasons, the use of green synthesized nanomaterials has considerably increased in the last 4 years (70).

Consequently, it would be useful to include a criterion addressing the two main nanomanufacturing approaches within the proposed classification system. This criterion would allow identifying NHPs which, due to their nanomanufacturing approach, need a specific focus when assessing their environmental risk. The question this criterion addresses would be: *what is the approach for the NHP nanomanufacturing?* The possible answers to this question would be either a top-down or bottom-up approach.

### 3.2. Proposed classification system

#### 3.2.1. Classification system structure

The classification system proposed includes a total of four classification criteria: principal mode of action, chemical composition of the core, medical purpose and nanomanufacturing approach (Table 4). Furthermore, the order in which these criteria have been organized in the system follows this rational:

1. Targeting the qualification of the NHP based on its primary mode of action.
2. Defining a battery of studies to meet the quality and safety requirements.
3. Defining the strategy by which compliance with performance and/or efficacy requirements will be assessed.
4. Including green nanotechnology and occupational risk considerations.

**Table 4:** Criteria of the proposed NHP classification system.

Criteria	Question	Subdivisions
Principal mode of action	Does the product exert its primary mode of action based on pharmacological, immunological or metabolic means?	NHPs with a pharmacological, immunological or metabolic primary mode of action NHPs without a pharmacological, immunological or metabolic primary mode of action
Chemical composition of the core	What is the chemical composition of the NHP?	Dispersion colloids Ceramic Metallic Semi-metallic Molecular colloids Polymer (natural/synthetic molecular building block) Associated colloids Carbon-based (with or without self-assembling properties)
Medical purpose	What kind of medical use does the NHP have?	Pharmacological: Pharmacological treating use Pharmacological prophylactic use Non-pharmacological: Physico-mechanical therapeutic (treating/prophylactic) Diagnostic use Galenic use
Nanomanufacturing approach	What is the approach for NHP nanomanufacturing?	Bottom-up Top-down

**Figure 4:**
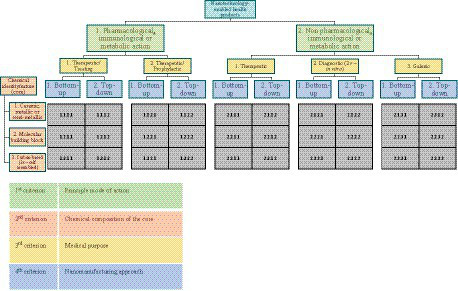
Proposed nanotechnology-enabled health products classification system.

Figure 4 shows the structure of the classification system.

The scope of the proposed classification system is intended to include both NHPs, as well as the corresponding regulatory documents that address relevant aspects to be considered during development for achieving regulatory approval of such products. Based on the latter, regulatory documents are classified in the compiled regulatory database according to the NHP they are referring to.

#### 3.2.2. Coding system and signatures

The proposed classification uses a coding system to reference each of the possible NHP categories. As shown in Figure 4, each subdivision in the classification is associated with an alphanumeric code. For a given NHP, each of the classification criterion is applied and the corresponding code established. At the end of the process, a distinctive 4-digit signature, namely the classification signature, is obtained to identify the NHP medical product according to the 4 criteria. For example, for a theoretical NHP with a non-pharmacological effect, whose core consists of silver nanoparticles, which has a diagnostic action *in vivo* and which is manufactured based on a bottom-up approach, the applicable signature would be 2.1.2.1 (Table 5A). Another practical example, for Doxil^®^, the first FDA-approved nanomedicine for which nanoliposomal drug carrier was used, applicable signature would be 2.3 s.3.1 (Table 5B) (29).

**Table 5:** Examples of classification assessment for two nanotechnology-enabled health products.

A			
Example	Criteria	Question	Subdivisions
Theoretical NHP with a non-pharmacological effect, whose core consists of silver nanoparticles, which has a diagnostic action *in vivo* and which is manufactured based on a bottom-up approach	Principal mode of action	Does the product exert its primary mode of action based on pharmacological, immunological or metabolic means?	2. NHPs with a non-pharmacological (broad sense) primary mode of action
	Chemical composition	What is the chemical composition of the NHP?	1. Ceramic, metallic or semi-metallic
	Medical purpose	What kind of medical use does the NHP have?	Non-pharmacological: 2. Diagnostic *in vivo*
	Nanomanufacturing approach	What is the approach for NHP nanomanufacturing?	1. Bottom-up
B			
Doxil^®^‘s nanoliposomal carrier	Principal mode of action	Does the product exert its primary mode of action based on pharmacological, immunological or metabolic means?	2. NHPs with a non-pharmacological (broad sense) primary mode of action
	Chemical composition	What is the chemical composition of the NHP?	3s. Carbon-based self-assembled
	Medical purpose	What kind of medical use does the NHP have?	Non-pharmacological: 3. Galenic action
	Nanomanufacturing approach	What is the approach for NHP nanomanufacturing?	1. Bottom-up

As indicated in the previous section, this classification system can also be applied for classifying regulatory documents, depending on their main scope or specific NHP category they relate to. In case a regulatory document does not meet one of the classification criteria, this can take the value “0” in the corresponding position of the signature. As examples:

- *EMA reflection paper on surface coating: general issues for consideration regarding parenteral administration of coated nanomedicine products* (47). This reflection paper is addressing general issues to consider during the development of nanomedicines that include a covalent or non-covalent coating. Consequently, this document is focused on the nanocoating of nanomedicines that may have an impact in the product pharmacokinetics and bio-distribution. No information excluding or limiting the scope of this document per chemical composition or nanomanufacturing approach has been identified. Thus, the corresponding signature of this document would be 2.0.3.0, meaning:

- 2.0.3.0

- *2 – non-pharmacological principle mode of action*

- *0 – chemical composition not defined or not limited*

- *3 – galenic action*

- *0 – nanomanufacturing approach not defined or not limited*

- *Joint MHLW/EMA reflection paper on the development of block copolymer micelle medicinal products* (45). This reflection paper addresses basic information for the pharmaceutical development of block-copolymer micelle nanomedicines. Active substances are incorporated in polymeric micelles that modify their pharmacokinetics, stability, and distribution. Nanomanufacturing is based on chemical procedures, therefore based on a bottom-up approach. Consequently, the corresponding signature of this document would be 2.3.2.1, meaning:

- 2.2.3.1

- *2 – non-pharmacological principal mode of action*

- *2 – block-copolymer core composition*

- *3 – galenic action*

- *1 – nanomanufacturing based on a bottom-up approach*

Finally, in the case of NHPs or regulatory documents that comply with more than one possibility for one criterion, the proposed system allows assigning two consecutive codes corresponding to the two applicable subdivisions. Theranostic agents are a common example of this situation. These are products with a dual activity, typically a diagnostic action combined with a pharmacological treating action (30, 31). In these cases, the first and third criteria (primary mode of action and intended medical purpose) would be twofold in the distinctive signature, i.e., (1.2).X.(1.2).X, where “X” corresponds to the code of the second and forth criteria that will depend on the chemical composition and the nanomanufacturing approach, respectively.

#### 3.2.3. Implementation of the classification system

The proposed classification system has been implemented to monitor current state of the art for regulatory documents pertaining to NHPs. With this aim, a database on regulatory documents has been created. This database includes regulatory documents, both published and under development until May 2023, from different regulatory stakeholders: competent authorities (EMA and FDA), working groups (EU Science Hub and SCENHIR) and standards issuing bodies (OECD, ISO, and ASTM). As a whole, this database reflects current knowledge in the regulation of NHPs and has therefore been considered as the NHP’s regulatory state of the art for the purposes of this work (32).

All regulatory references included in said database are compiled in Table 6 based on their document typology (hazard testing, legislative document, paper, guideline, report or standard) and document version (either current version or under development). Additionally, the number of defined criteria within the classification signature of each document (i.e., the number of criteria not identified by code “0” in the classification signature) is also included. The classification signature of a regulatory document is not only reflecting the specific NHP category the document refers to but also could be indicative of whether a document is general in scope or specific to a given NHP category. For example, it is assumed that the scope of a regulatory document with classification signature ‘0.0.0.0’ is wider than that of a document with signature 1.1.1.1. In the first case there is no limit regarding any of the classification criteria whereas in the second case that document would be specific only to metallic nanomaterials with pharmacological principal mode of action, treating action and nano manufactured based on a bottom-up approach.

**Table 6:** Documents from the regulatory database classified based on document type (A), number of defined criteria of the classification system and version (B).

A	
Document type	Number of documents
Hazard testing	3
Legislative document	5
Paper	21
Guideline	20
Report	25
Standard	192

B				
Number of defined criteria	Current version	Current version (%)	Under development	Under development (%)
0	139	58.4	12	41.38
1	47	19.75	7	24.14
2	34	14.29	3	10.34
3	3	1.26	1	3.45
4	15	6.3	6	20.69
	238	100	29	100

(A) Regulatory documents are classified based on their document typology: hazard testing (protocols for assessing biological safety), legislative documents, papers from regulatory bodies, guidelines, reports, standards from ISO and ASTM. (B) Regulatory documents are classified based on their number of defined criteria of the proposed classification system and the version (i.e., current version or under development).

**Figure 5:**
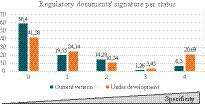
Percentage of regulatory documents according to their number of defined criteria. Regulatory documents listed in the created regulatory database have been classified according to the number of defined criteria as an indicator of their degree of specificity. This means that regulatory documents with none of their classification criteria defined are general, non-specific with regards to NHPs. On the side, regulatory documents with all their classification criteria defined are specific as they address NHPs with concrete features as defined according to the classification system.

Next, Table 6 results have been represented in Figure 5. When classifying regulatory documents either currently published or under development, there are differences in relation to the number of defined criteria. For currently published documents, there is a tendency toward a higher number of undefined classification criteria, i.e., the number of published documents of a general scope (non-specific of a certain NHP type or application) is higher than for the documents under development. On the contrary, in this latter case there is a tendency toward regulatory documents with a more specific scope.

This scenario of ‘regulatory science specialization’ resembles a similar situation taking place, at a greater advantage, for advanced therapy medicinal products (ATMPs). The assessment of ATMPs for regulatory approval requires very specific expertise, which went beyond the traditional pharmaceutical field and covers areas bordering on other sectors such as biotechnology, nanotechnology itself and medical devices (33). In response, the EMA created a specialized Committee for Advanced Therapies (CAT) in 2009 to review the marketing authorization applications for these complex medicinal products (34) and to guide ATMPs developers to facilitate the product access to the patients. However, after more than 15 years, ATMPs have gained wider recognition and are now more commonly utilized. In accordance with the proposal for a Medicinal Product Regulation published by the EC, there are indications that the CAT may be discontinued but the expertise retained through various working groups (35). Likewise, the FDA Center for Biologics Evaluation and Research (CBER) was created in 1987 for ensuring compliance with safety and efficacy requirements of advanced therapies in the USA (36).

Coming back to NHPs, a regulatory state of the art becoming more specialized can be noticed (1). Regulatory bodies must shift from their approach of considering nanoparticles as small versions of larger molecules to one where they recognize their fundamental different properties (78, 79). This is in line with a report published by the OECD in 2022 on important issues on the risk assessment of manufactured nanomaterials. According to this report, while generalization in methodologies is preferred, for nanomaterials a case-by-case, i.e., specific, approach is envisioned (80). In this scenario, the proposed classification system will be a useful tool for regulators to monitor how regulatory science is developing the understanding of NHPs applications. Additionally, also similar to some initiatives for ATMPs such the ARDAT EU funded project (81), this classification system could be useful for developing prospective regulatory databases that help manufacturers and developers link their NHPs with the applicable guidelines.

## 4. Conclusion

The proposed classification system could allow regulators to monitor the regulatory state of the art of NHPs for performing strategic regulatory development. By using future-oriented methodologies such as Horizon Scanning, it would be feasible to elucidate the main trends in the future development of NHPs. By mapping the classification signatures of these future trends with the regulatory state of the art, it will be possible to foresee the existence of possible gaps in regulation that will hamper the regulatory approval of these developments. Regulators will be able to anticipate these future regulatory needs and target the development of guidelines and standards in an efficient manner.

Furthermore, this classification system could be also useful for developers and manufacturers to link their technologies with the applicable regulatory guidelines. By applying this classification system to the regulatory state of the art and integrating it in a platform, developers could query on their NHPs with a specific classification signature. The platform would return all the regulatory guidelines potentially applicable to that technology, from the most general to the most specific documents. All in all, the potential uses of the proposed classification system are aimed to foster NHPs development, NHPs regulatory approval and, consequently, mitigate current *valley of death*.

## Data availability statement

The raw data supporting the conclusions of this article will be made available by the authors, without undue reservation.

## Author contributions

FDRG conceptualization, methodology, formal analysis, investigation, data curation, writing—original draft, visualization, and project administration. PRG, DM, and OP: conceptualization, validation, writing—review and editing, supervision, project administration, and funding acquisition. All authors contributed to the article and approved the submitted version.

## Funding

This work was supported by the Industrial Doctorates Plan of the Department of Research and Universities of the Generalitat de Catalunya (Grant n° 202015).

## Conflict of interest

Authors FDRG, OP, and DM were employed by company Asphalion SL.

The remaining author declares that the research was conducted in the absence of any commercial or financial relationships that could be construed as a potential conflict of interest.

## Publisher’s note

All claims expressed in this article are solely those of the authors and do not necessarily represent those of their affiliated organizations, or those of the publisher, the editors and the reviewers. Any product that may be evaluated in this article, or claim that may be made by its manufacturer, is not guaranteed or endorsed by the publisher.

## Footnotes

^1^‘Nanotechnology-enabled health product’ is an overarching term for nanomedicines (health products including nanomaterials that are regulated as medicinal products) and nanomedical devices (health products including nanomaterials that are regulated as medical devices).

^2^Cooperative Patent Classification (CPC) A61 applies for ‘*medical or veterinary science; hygiene*’.

^3^Regulatory science is defined by the EMA as ‘*a range of scientific disciplines that are applied to the quality, safety and efficacy assessment of medicinal products and that inform regulatory decision-making throughout the lifecycle of a medicine. It encompasses basic and applied medicinal science and social sciences, and contributes to the development of regulatory standards and tools’.*
